# Water status and macronutrient concentrations, but not carbon status, of *Viscum album* ssp. *album* are determined by its hosts: a study across nine mistletoe–host pairs in central Switzerland

**DOI:** 10.3389/fpls.2023.1142760

**Published:** 2023-05-08

**Authors:** Ao Wang, Arun K. Bose, Marco M. Lehmann, Andreas Rigling, Arthur Gessler, Longfei Yu, Maihe Li

**Affiliations:** ^1^ Institute of Environment and Ecology, Tsinghua Shenzhen International Graduate School, Tsinghua University, Shenzhen, Guangdong, China; ^2^ Forest Dynamics, Swiss Federal Institute for Forest, Snow and Landscape Research WSL, Zürcherstrasse Birmensdorf, Switzerland; ^3^ Institute of Terrestrial Ecosystems ITES, Swiss Federal Istitute of Technology, ETH Zürich, Universitätstrasse 16 Zurich, Switzerland; ^4^ Forestry and Wood Technology Discipline, Khulna University, Khulna, Bangladesh; ^5^ Key Laboratory of Geographical Processes and Ecological Security in Changbai Mountains, Ministry of Education, School of Geographical Sciences, Northeast Normal University, Changchun, Jilin, China; ^6^ College of Life Science, Hebei University, Baoding, Hebei, China

**Keywords:** macronutrient, mistletoe-host pair, non-structural carbohydrate (NSC), *Viscum album* ssp. album, water availability

## Abstract

**Introduction:**

European mistletoe, *Viscum album* L., is a hemiparasite that can infect various tree species, yet our understanding of its physiological interactions with host species is limited.

**Methods:**

Nine mistletoe–host pairs (i.e. *V. album* ssp. *album* growing on nine different broadleaf tree species) under different growth conditions in central Switzerland were selected to examine the carbon, water and nutrient relationships between mistletoe and its hosts. We measured leaf morphological traits, isotopic compositions (δ13C and δ15N), concentrations of non-structural carbohydrates (NSC) and specific compounds (i.e. mobile sugars and starch), and macronutrients (i.e. N, P, K, Ca, Mg, S) in leaf and xylem tissues of both mistletoe and its hosts.

**Results and Discussion:**

There were only non-significant relationships between NSC concentrations in mistletoe and in its host species across the nine mistletoe–host pairs, suggesting the carbon condition of *V. album* ssp. *album* is determined by both the heterotrophic carbon transfer and self-photosynthetic capacity among different mistletoe-host pairs. However, mistletoe leaf morphological traits (single leaf area and mass, and leaf mass per unit leaf area) did not change across the nine mistletoe–host pairs, and mistletoe leaf δ13C, water content and macronutrient concentrations were linearly correlated with those in the host leaves. Macronutrients showed accumulations in mistletoe across the nine pairs. Further, tissue N concentrations were significantly higher in mistletoe grown on N-fixing hosts than on non-N-fixing hosts. Finally, leaf N:P in mistletoe was significantly correlated with the ratio in the host across the nine mistletoe–host pairs. Overall, our results indicate strong relationships between mistletoe and its hosts for water- and nutrient-related traits, but not for carbon-related traits, which demonstrates that *V. album* ssp. album can adjust its physiology to survive on different deciduous tree species hosts and under different site conditions.

## Introduction

*Viscum album* L., known as European mistletoe, is widely distributed in central Europe (Becker, 2000). This species has four subspecies with a similar appearance, such as green leaves and haustoria connected to the xylem tissue of the host branch (Zuber, 2004). *Viscum album* ssp. *album* is the only subspecies that can infect multiple broadleaf host tree species, suggesting that it has potential to enlarge its distribution range in different environmental habitats (Zuber and Widmer, 2009).

Unlike coniferous hosts, broadleaf host trees often differ considerably in morphological appearance, as well as in photosynthesis and transpiration capacities, which may lead to a more complex mistletoe–host relationship (Ullmann et al., 1985; LuÈttge et al., 1998; Krasylenko et al., 2020). Existing research about mistletoe infection has been concentrated on the effects of mistletoe on the host plants (Escher et al., 2004a; Yan et al., 2016), while less attention has been paid to the paired mistletoe–host relationship (Urban et al., 2012; Mutlu et al., 2016, Bilgili et al., 2020). Hence, very little information exists on the general patterns of the relationships between the non-host-specific mistletoe and its various hosts (Le et al., 2016b, Scalon and Wright, 2017).

Carbon, water and nutrients are the three most fundamental elements for understanding the mechanisms and relationships between mistletoe and its host (Glatzel, 1983; Schulze et al., 1984). Several hypotheses have been proposed to interpret the physiological mechanisms of the mistletoe–host relationship, i.e. the C-parasitism hypothesis, N-parasitism hypothesis, and mimicry hypothesis (Schulze et al., 1984; Scalon et al., 2013). The C-parasitism hypothesis suggest that heterotrophic carbon demand from the host is the limiting factor for the growth of mistletoe (Schulze et al., 1984; Wang et al., 2008; Těšitel et al., 2010). Anatomical analysis has indicated that no phloem connection exists between *V. album* and its host, which is a unique characteristic for this species and suggests that no carbon transfer occurs through phloem sap from the host to the mistletoe tissues (Sauter, 1980; Pate and Atkins, 1983; Smith and Gledhill, 1983). In line with the C-parasitism hypothesis, however, in some model-based studies using the difference between observed and theoretical δ^13^C values in mistletoe leaves, only a portion of the carbon in mistletoe was found to be produced through its own photosynthesis activities, while a significant amount (up to 80%) was absorbed heterotrophically (Pate et al., 1991; Popp and Richter, 1998; Wang et al., 2008). Further, Escher et al. (2004b) indicated that *V. album* can acquire organic heterotrophic carbon from the host in the form of xylem-mobile organic acids and amino acids, suggesting the possibility of carbon uptake through xylem-flow to support its growth demand (Marshall and Ehleringer, 1990; Escher et al., 2004a; Escher et al., 2004b; Těšitel et al., 2010).

Compared with the uncertain carbon relationship, *V. album* – with a lower water potential and higher transpiration rate compared with the host – relies completely on continuous water uptake from the host to meet its water demand (Schulze et al., 1984; Zuber, 2004; Glatzel and Geils, 2009). Thus, water is a key factor determining the mistletoe–host relationship (Schulze et al., 1984; Zweifel et al., 2012; Scalon and Wright, 2015). Previous studies showed that δ^13^C values in mistletoe leaves were significantly lower but leaf water content was generally higher than in the host leaves, indicating a lower water use efficiency (WUE, ratio of photosynthetic rate to transpirational water loss) but a higher water uptake ability of mistletoe compared with its hosts (Schulze et al., 1984; Wang et al., 2021; Wang et al., 2022). Zweifel et al. (2012) investigated the water relationship between pine mistletoe (*V. album* ssp. *austriacum*) and its host Scots pine (*Pinus sylvestris*). They concluded that mistletoe, in contrast to its host, barely regulates the closure of its stomata in response to drought. Such strategies of hemiparasites indicate a compensation mechanism for the additional water loss from mistletoe for its host Scots pine, to survive but also to avoid reducing the carbon assimilation of the host in drought conditions.

Similar to water uptake, due to its lack of a root system *V. album* also relies completely on absorption from host tissues for nutrient uptake (Smith and Gledhill, 1983; Glatzel and Geils, 2009). Mistletoe continuously absorbs nutrients from the host tissues, which can result in nutrient accumulation in mistletoe, especially for macronutrient elements (i.e. N, P, K), compared with in its hosts (Ture et al., 2010; Mutlu et al., 2016). The main reason for this effect is the absence of a phloem connection between mistletoe and its host (Smith and Gledhill, 1983), as accumulated nutrients cannot be reallocated through phloem sap flow (Bell and Adams, 2011; Lo Gullo et al., 2012). For nitrogen, for example, an N-parasitism hypothesis has been proposed to interpret the N-flow mechanisms between mistletoe and its host (Pate and Atkins, 1983; Schulze and Ehleringer, 1984). It proposes that mistletoe is more strongly limited than the host by the concentration of available nitrogen and that relatively high transpiration rates help it to extract sufficient N from the host xylem stream (Glatzel and Geils, 2009; Scalon and Wright, 2015). However, no consistent evidence has been found to support this hypothesis (Schulze et al., 1991; Marshall et al., 1994), and the N relationship seems to vary with both mistletoe and host identity, habitat, and possibly also with the N-fixing ability of the host species.

Moreover, previous studies were mainly focused on the absolute concentrations of nutrients in mistletoe and/or in its hosts (Lo Gullo et al., 2012; Mutlu et al., 2016), rather than on the nutrient relations between mistletoe and its hosts. Stoichiometric stability has been regarded as a strategy of plants to balance nutrient allocation and transfer (Andersen et al., 2004; Moe et al., 2005). The stoichiometry in vascular plants is found to be significantly correlated with the soil nutrient conditions (Yu et al., 2011; Sun et al., 2021). However, the stoichiometric stability of hemiparasite plants, which are detached from the soil, is still uncertain (Tang et al., 2019). Specifically, little is known about the stoichiometry between mistletoe and its hosts, i.e. in a relationship where nutrient uptake is unidirectional from the host to the mistletoe and driven only by transpiration processes. Moreover, even though mistletoe may not directly absorb carbon resources from its host (Wang et al., 2022) and can maintain stable levels of non-structural carbohydrates (NSCs), the water availability-dependent nutrient absorption rate and the variability across host species are likely to lead to variations in nutrient levels in mistletoe tissues (Chen et al., 2013; Wang et al., 2021; Wang et al., 2022), leading to changes in the ratios between NSCs and macronutrients. Hence, the stoichiometry related to NSCs, N and P in mistletoe leaves can be affected by the water and nutrient availability of its host, as well as by its own regulation mechanism linked with its own physiological processes.

To better understand the carbon, water and nutrient relationships across various host species, we conducted a summertime field sampling assessment of European mistletoe (*V. album* ssp. *album*) growing on nine broadleaf host species in different growth conditions. We sampled and analyzed tissues from nine mistletoe–host pairs in terms of morphological (i.e. single leaf area, single leaf mass, leaf mass per area), isotopic (i.e. δ^13^C, δ^15^N), and physiological variables associated with carbon (i.e. total NSCs and individual compounds), water (i.e. leaf water content), and macronutrients (i.e. N, P, K, Ca, Mg, S). We tested the following hypotheses:

H1: The status of available carbon (i.e. total NSCs and individual compounds) in *V. album* is independent of its hosts’ carbon status and growth conditions, due to the special mechanisms of obtaining carbon resource from heterotrophic and autotrophic pathways.

H2: The leaf water content and water use efficiency (WUE, indicated by δ^13^C) of *V. album* are correlated with values in host leaves because mistletoe needs to maintain a stable water potential gradient between the host and itself to guarantee a unidirectional water transfer.

H3: The macronutrient concentrations and the stoichiometry of NSCs, N and P in mistletoe tissues are positively correlated with values in host trees.

H4: The N concentration in mistletoe growing on N-fixing hosts is higher than in mistletoe growing on non-N-fixing host trees, due to continuous nutrient transfer from the host to the mistletoe tissues.

## Material and methods

### Study sites and sampling

**Figure 1:**
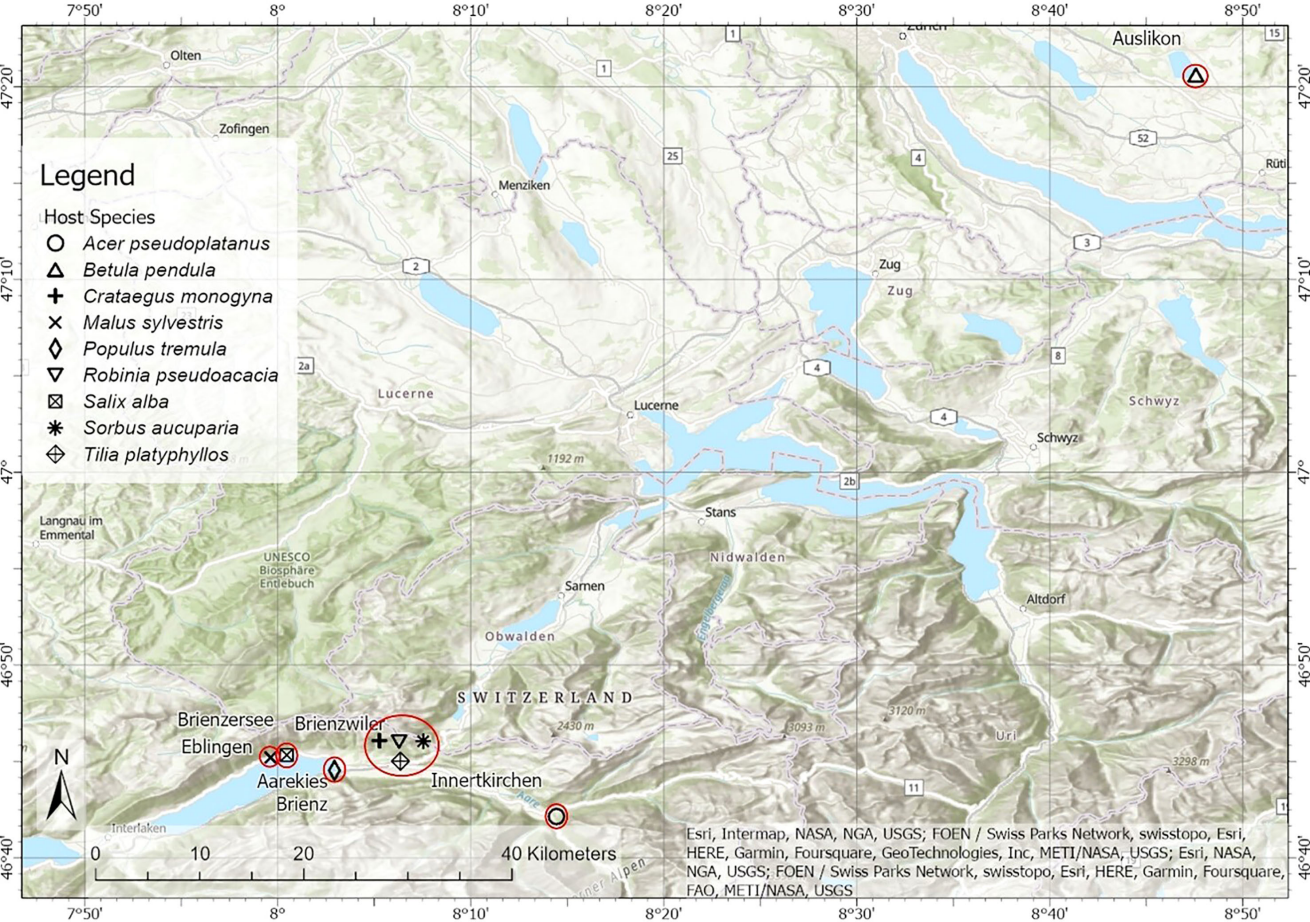
Map of the sampling sites for the nine mistletoe–host pairs in central Switzerland. Different symbols indicate the nine different pairs and red circles indicate the sampling sites (locations). The appended picture in the middle magnifies the sampling site which is on the bottom of the map.

From July 9^th^ to July 11^th^, 2019, samples were collected from six sites with different habitats in central Switzerland, where many deciduous trees are infected by European mistletoe (*Viscum album* ssp. *album*; **Figure 1**; **Table S1**). Nine co-grown pairs of mistletoe and broadleaf tree species were sampled. Among these species, five pairs (i.e. *Acer pseudoplatanus, Tilia platyphyllos, Crataegus monogyna, Robinia pseudoacacia, Sorbus aucuparia*) were sampled in closed forests on a south-facing slope close to Innertkirchen (46°42’32” N, 8°14’29” E) and Brienzwiler (46°45’4” N, 8°5’55” E). *Viscum Album*–*Malus sylvestris* was sampled on a south-facing slope in Ebligen (46°45’13” N, 7°59’38” E). Directly along the banks of Lake Brienz, samples were collected of *V. album*–*Salix alba* pairs (46°45’20” N, 8°0’28” E) and *V. album*–*Populus tremula* pairs (46°44’32” N, 8°2’56” E). *Viscum Album*–*Betula pendula* samples were collected directly along the banks of Lake Pfäffiker close to Auslikon (47°20’37” N, 8°47’34” E). All the sampled trees were found severely infected by mistletoes, which has at least six mature mistletoe clusters penetrating into the branches. Four to six host trees infected by mistletoe were found for each host species, and a randomly selected mistletoe–host branch was cut from each selected host tree (n = 4–6) with long pruning shears. All the mistletoe clusters from the sampled trees were fully matured by counting the number of internodes since mistletoe grows with one node of branch per year. The samples included leaves and twigs (only current-year tissues) of both mistletoe and its hosts. The twig phloem and twig xylem were separated immediately after sampling, and only the xylem was stored in an ice box and used for future laboratory analysis. All harvested tissues of both mistletoe and host trees were stored in an ice box in the field, then dried at 65°C to constant weight. After drying, each sample was ground to a fine and homogeneous powder with a Retsch MM 300 ball mill (Retsch, Haan, Germany).

### Analysis of leaf traits for mistletoe and different host species

For each mistletoe–host pair, at least three host leaves and eight mistletoe leaves were randomly selected for leaf trait measurements. The fresh mass of all leaves was first measured, and then dry leaf mass was measured after oven-drying the samples at 65°C for 5 d. Leaf water content (LWC) was calculated on a fresh mass basis as:


Eqn. 1 $$\text{LWC}=\frac{Fresh\;leaf\;mass\;\left(mg\right)-dry\;leaf\;mass\;\left(mg\right)}{Dry\;leaf\;mass\;\left(mg\right)}\;\times 100\;$$

Leaf dry mass per unit leaf area (LMA) was then calculated as:


eqn. 2 $$\text{LMA}=\frac{Dry\;leaf\;mass\;\left(mg\right)}{leaf\;area\;\left(cm^{2}\right)}\;\;\;\;\;$$

where the leaf area of each mistletoe–host pair (**Figure S1**) was measured using a scanner and image analysis software (PIXSTAT v1.3, WSL, Birmensdorf, Switzerland).

### Analysis of nutrient elements

The prepared ground and dried plant material (0.5 g) was dried again at 65°C for another 12 h. HNO_3_ (8_ ml_, 65%) was added to the ground samples, and the samples were then heated with microwave technology (imUltraclave IV, MLS GmbH, Leutkirch im Allgäu, Germany). The temperature of the microwave was gradually increased to 175°C over 20 min total. The samples and chemicals were filtered with Whatman filters into 50-ml sterile tubes and diluted to 50 ml with ultra-pure water for ICP-OES analysis. Before the ICP-OES analysis, standards were prepared using a 1000 ppm multi-element solution. Nutrient element (i.e. N, P, K, Ca, Mg, S, Fe, Mn, Al, Zn) measurements were conducted with an Optima 7300DV (Perkin Elmer Inc., Shelton, CT, USA) after calibration using the standards (Rezić and Steffan, 2007).

### Analysis of total non-structural carbohydrates and individual compounds

NSCs were defined as low-molecular-weight sugars and starch, and analysis followed the protocol used by Schönbeck et al. (2018). About 10 mg of the sample powder was first vortexed with 2 ml deionized water and then boiled in the steam for 30 min. For free sugar analysis, a 200 μl aliquot of the extract was treated with invertase and isomerase (in 0.4 M Na-acetate buffer; Sigma-Aldrich, St Louis, MO, USA) to break down sucrose to fructose and glucose. For total NSC analysis, a 500 µl aliquot of the extract (including sugars and starch) was incubated with a fungal amyloglucosidase from *Aspergillus niger* (Sigma-Aldrich) for 15 h at 49°C to digest starch into glucose. Both free sugars and total NSC concentrations were determined at 340 nm in a 96-well microplate photometer (Multiskan GO, Thermo Fisher) after enzymatic conversion of glucose molecules derived from sugars and starch to gluconate-6-phosphate (via isomerase, hexokinase and glucose-6-P dehydrogenase; all supplied by Sigma-Aldrich). NSC concentrations were expressed as a percentage of dry matter, and the concentration of starch was calculated as total NSCs minus free sugars.

### Analysis of ^13^C and ^15^N abundance

Around 1 mg of ground tissue was weighed into tin cups. Organic carbon and nitrogen were converted to CO_2_ and N_2_ in a Euro EA3000 elemental analyzer (Hekatech GmbH, Wegberg, Germany) connected to an isotope ratio mass spectrometer (IRMS; Delta V Advantage, Thermo Fisher Scientific, Bremen, Germany) to determine the total carbon and nitrogen concentrations, as well as the isotopic composition (δ^13^C, δ^15^N) of both elements. Laboratory standards with known δ^13^C and δ^15^N values were measured with a precision of 0.1‰. The isotope ratios in all samples were expressed using the δ notation (‰) relative to the international standard Vienna Pee Dee Belemnite (VPDB) (for δ^13^C) and standard atmosphere N concentration (for δ^15^N).

### Data analysis

The effects of site and host species were analyzed separately, as all mistletoe–host pairs did not necessarily occur in every study site. For each parameter, a linear mixed-effects modeling approach without random effects was first applied. And then the linear mixed-effects model considering each variable as a fixed effect and the mistletoe–host pair within the different sampling sites as random effects was also applied to make a comparison. Fixed effect variables were log-transformed (if needed) to meet assumptions of normality of the residuals and homogeneity of the variances. The model output showed that random effects (i.e. site and mistletoe-host pair) truly affected the results for almost all variables (**Tables S3**, **S4**). In parallel, one-way analysis of variance (ANOVA) was performed to compare the means among the nine mistletoe–host pairs and among the six different sampling sites for different tissue types (i.e. host leaf, host xylem, mistletoe leaf, mistletoe xylem). A *post-hoc* (Tukey-HSD) analysis was then performed to compare differences among tissues regardless of host species and site effects. Pearson correlations were performed to study the relationship between mistletoe and host traits. Standardized major axis (SMA) slopes (Warton et al., 2006) were used to match the best fit proportional relationship of traits between mistletoe and its hosts. R version 4.1.0 was used for all statistical analyses (R Core Team, 2021).

## Results

### NSC concentrations

**Figure 2:**
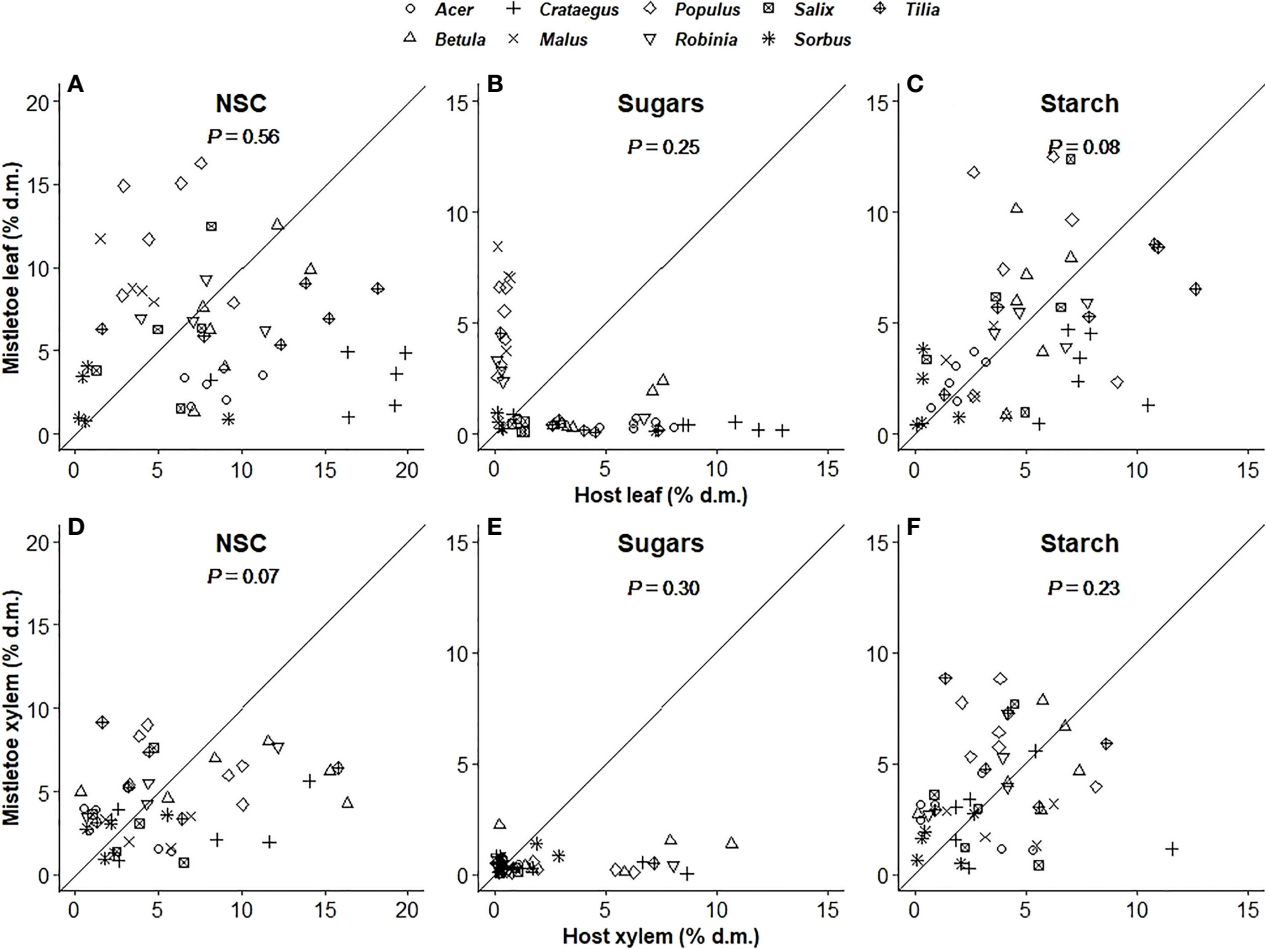
Relationship between mistletoe (*Viscum album* ssp. *album*) and its host for sugar, starch and total non-structural carbohydrate (NSC; sum of sugars and starch) concentrations (% dry matter) in leaf **(A–C)** and xylem **(D–F)** tissues across nine mistletoe–host pairs in central Switzerland (indicated by different symbols). No significant relationships were observed (P-values of correlation analyses are given). The black solid line denotes the 1:1 line.

The concentration of total NSCs and of individual compounds varied significantly with host identity in host leaves but not in host xylem (**Table 1**). In mistletoe, however, NSC concentrations in both leaves and xylem varied significantly across the nine mistletoe–host pairs, except for sugars in mistletoe xylem (**Table 1**). The concentration of total NSCs and of individual compounds in mistletoe were not correlated with those in its host (**Figure 2**), and values tended to be lower in mistletoe than in its host within each tissue type (**Table 2**; **Figure 2**).

**Table 1:** One-way ANOVA results for the effects of mistletoe (*Viscum album* ssp. *album*)–host pairs (*n*=9) on different variables: Total non-structural carbohydrate (NSC), sugar and starch concentrations, single leaf area and mass, leaf dry mass per unit leaf area (LMA), leaf water content, nutrient concentrations (nitrogen [N], phosphorus [P], potassium [K], calcium [Ca], magnesium [Mg] and sulfur [S]), and element stoichiometry in leaves and xylem tissues of mistletoe and host species.

	Variable	DF	F-value host leaf	F-value mistletoe leaf	F-value host xylem	F-value mistletoe xylem
**Available carbon** **(% dry matter)**	**NSC**	8	16.4***	9.8***	1.3	6.1***
	**Sugars**	8	20.5***	28.8***	1.4	2.1
	**Starch**	8	7.4***	3.4**	1.7	5.8***
**Isotope ratio (‰)**	**δ^13^C**	8	24.1***	12.3***	15.6***	10.4***
	**δ^15^N**	8	17.4***	71.8***	142.7***	37.1***
**Single leaf area (cm^2^)**		8	43.5***	1.5	NA	NA
**Single leaf mass (g)**		8	4.6***	2.5	NA	NA
**LMA (g cm^-2^)**		8	13.8**	1.2	NA	NA
**Leaf water content (%)**		8	11.2***	9.8***	NA	NA
**Nutrient** **concentrations** **(mg g^-1^)**	**N**	8	69.7***	8.6***	54.7***	8.8***
	**P**	8	6.7***	6.2***	10.2***	6.2***
	**K**	8	21.8***	5.8***	12.8***	5.2***
	**Ca**	8	19.1***	2.8*	24.1***	6.5***
	**Mg**	8	18.3***	11.0***	4.1***	22.0***
	**S**	8	25.9***	11.3***	10.9***	6.6***
**Stoichiometry**	**NSC:N**	8	12.8***	10.6***	1.4	8.4***
	**NSC:P**	8	17.1***	8.9***	2.1	6.2***
	**N:P**	8	15.8***	9.2***	10.5***	10.4***

*P<0.05 **P<0.01 ***P<0.001.

The number of degrees of freedom (DF) and F-values are given.NA: No data for xylem tissue.

**Table 2:** Concentrations of total non-structural carbohydrate (NSC), sugar and starch concentrations, single leaf area and mass, leaf dry mass per unit leaf area (LMA), leaf water content, nutrient concentrations (nitrogen [N], phosphorus [P], potassium [K], calcium [Ca], magnesium [Mg] and sulfur [S]), and element stoichiometry in leaf and xylem tissues across nine mistletoe (*Viscum album* ssp. *album*)–host pairs in central Switzerland.

	Variable	Host leaf	Mistletoe Leaf	Host Xylem	Mistletoe Xylem	ML: HL	MX: HX
**Available** **carbon (% dry matter)**	**NSC**	8.2 ± 0.8^a^	6.3 ± 0.6^a^	5.4 ± 0.6^b^	4.3 ± 0.3^b^	0.7 ± 0.1^A^	0.8 ± 0.1^A^
	**Sugars**	3.5 ± 0.5^a^	1.7 ± 0.3^b^	1.9 ± 0.4^b^	0.5 ± 0.1^c^	0.4 ± 0.03 ^A^	0.3 ± 0.01^A^
	**Starch**	4.7 ± 0.4^a^	4.5 ± 0.5^a^	3.5 ± 0.4^a^	3.8 ± 0.3 ^a^	0.9 ± 0.04^A^	1.0 ± 0.01^A^
**Isotope** **ratios (‰)**	**δ^13^C**	-29.7 ± 0.2^a^	-31.1 ± 0.2^b^	-29.3 ± 0.3^a^	-30.8 ± 0.3^b^	1.0 ± 0.01^A^	1.0 ± 0.01^A^
	**δ^15^N**	0.2 ± 0.3^b^	1.9 ± 0.3^a^	0.7 ± 0.2^b^	0.9 ± 0.3^b^	1.7 ± 0.4^A^	1.0 ± 0.03^B^
**Single leaf area (cm^2^)**		15.8 ± 1.7^a^	3.6 ± 0.2^b^	NA	NA	0.5 ± 0.04	NA
**Single leaf mass (g)**		0.2 ± 0.07^a^	0.1 ± 0.01^b^	NA	NA	0.5 ± 0.01	NA
**LMA (cm^2^ g^-1^)**		14.2 ± 0.8^a^	5.5 ± 0.1^b^	NA	NA	0.4 ± 0.04	NA
**Leaf water content (%)**		60.8 ± 1.7^a^	68.7 ± 0.6^a^	NA	NA	1.2 ± 0.04	NA
**Nutrient concentrations (mg g^-1^)**	**N**	20.6 ± 1.1^b^	31.9 ± 1.2^a^	8.1 ± 0.3^c^	27.6 ± 0.8^a^	1.6 ± 0.05^B^	3.7 ± 0.2^A^
	**P**	1.5 ± 0.1^b^	4.4 ± 0.1^a^	0.8 ± 0.1^c^	4.1 ± 0.1^a^	3.2 ± 0.1^B^	5.8 ± 0.3^A^
	**K**	11.1 ± 0.7^b^	27.4 ± 0.8^a^	5.3 ± 0.5^c^	23.4 ± 0.5^a^	3.1 ± 0.3^B^	6.0 ± 0.5^A^
	**Ca**	13.1 ± 0.6^a^	10.5 ± 1.5^b^	16.6 ± 1.2^a^	5.3 ± 0.3^c^	0.9 ± 0.1^A^	0.4 ± 0.03^B^
	**Mg**	2.2 ± 0.1^a^	1.9 ± 0.3^a^	0.8 ± 0.04^b^	2.5 ± 0.1^a^	1.0 ± 0.04^B^	3.5 ± 0.2^A^
	**S**	2.1 ± 0.6^a^	2.8 ± 0.7^a^	0.6 ± 0.2^b^	2.4 ± 0.4^a^	1.6 ± 0.04^B^	4.1 ± 0.2^A^
**Stoichiometry**	**NSC:N**	0.5 ± 0.05^a^	0.2 ± 0.03^b^	0.7 ± 0.1^a^	0.2 ± 0.02^b^	1.1 ± 0.2^A^	0.5 ± 0.1^B^
	**NSC:P**	6.9 ± 0.8^a^	1.7 ± 0.2^b^	8.7 ± 1.2^a^	1.1 ± 0.1^b^	0.6 ± 0.1^A^	0.3 ± 0.1^B^
	**N:P**	14.8 ± 0.5^a^	7.3 ± 0.2^b^	12.2 ± 0.9^a^	6.9 ± 0.2^b^	0.6 ± 0.1^A^	0.5 ± 0.02^A^

Different lowercase letters indicate significant differences among tissues, and different capital letters indicate significant differences between the mistletoe–host ratio of leaf (ML: HL) and xylem (MX : HX) tissues (one-way ANOVA and Tukey-HSD post-hoc test). Mean values ± 1 SE are given (n=48).

### Leaf morphological traits and water content

**Figure 3:**
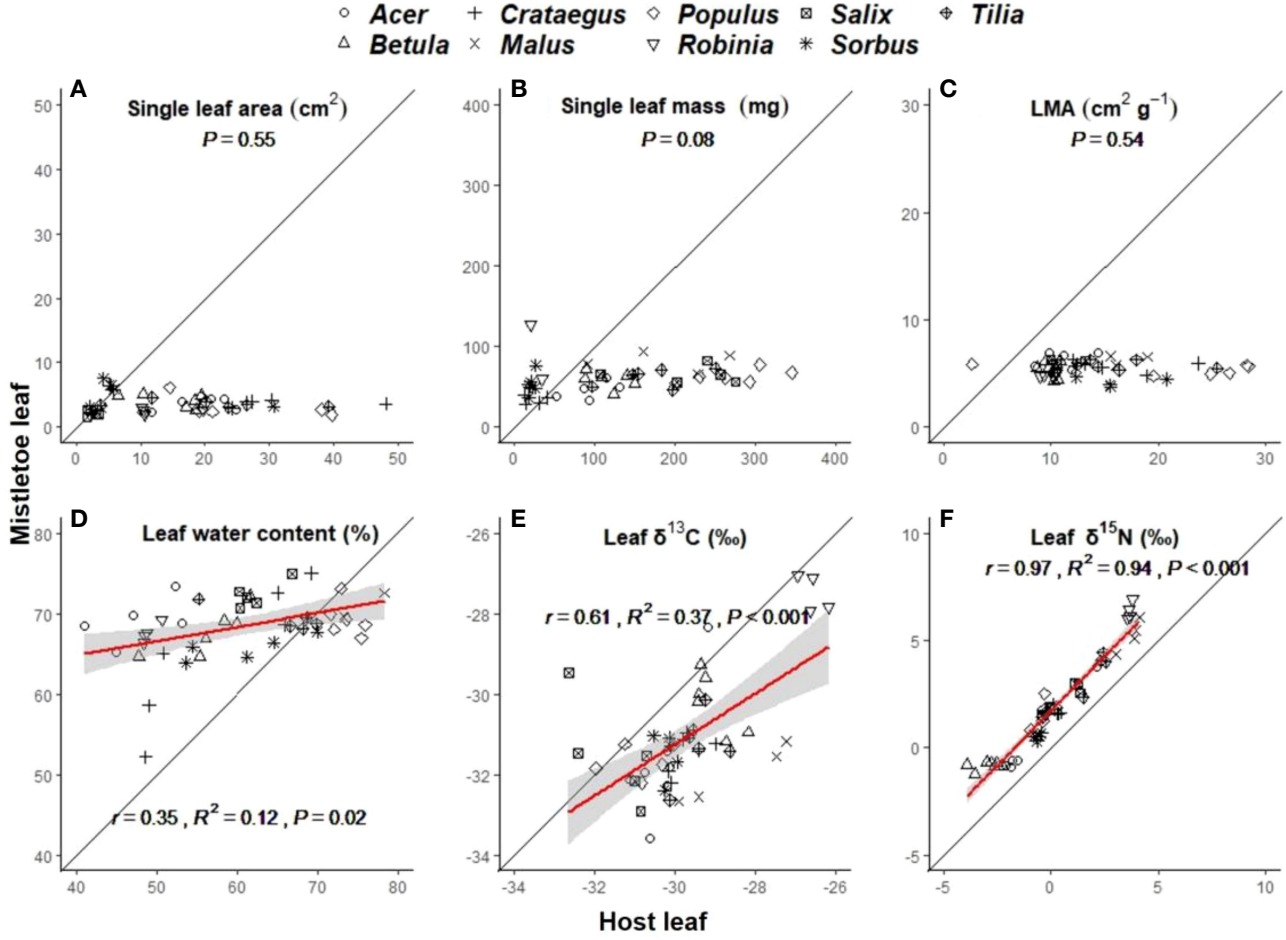
Linear relationships between mistletoe (*Viscum album* ssp. *album*) and its host for single leaf area **(A)**, single leaf mass **(B)**, leaf dry mass per unit leaf area (LMA) **(C)**, leaf water content **(D)**, leaf δ^13^C **(E)** and leaf δ^15^N **(F)** across nine different mistletoe–host pairs in central Switzerland (indicated by different symbols). The standardized major axis (SMA) and the 95% confidence interval are indicated with a red line and a gray band, respectively (where P<0.05). The black solid line denotes the 1:1 line. The x- and y- scale differs across the panels according to the values of each variable.

Leaf morphological traits (i.e. single leaf area, single leaf mass and LMA) in host leaves varied significantly with host identity (**Table 1**), while those in mistletoe leaves showed no difference across the nine mistletoe–host pairs (**Table 1**). No leaf morphological traits were correlated between mistletoe and its host, but the values were significantly lower for mistletoe leaves than for host leaves (**Table 2**; **Figures 3A–C**). Leaf water content showed significant variation in both mistletoe and host leaves across the mistletoe–host pairs (**Table 1**). There was a linear correlation between leaf water content in mistletoe and its host (r=0.35, R^2 ^= 0.12, P=0.02), and the mean value tended to be higher in mistletoe leaves than that in host leaves (**Table 2**; **Figure 3D**).

### Stable isotope ratios

δ^13^C and δ^15^N varied significantly across the nine mistletoe–host pairs in both mistletoe and host leaf and xylem tissues (**Table 1**). There were positive linear relationships between mistletoe and host leaves for δ^13^C (r=0.61, R^2 ^= 0.37, P<0.001) and δ^15^N (r=0.97, R^2 ^= 0.94, P<0.001) among the mistletoe–host pairs (**Figures 3E, F**). δ^13^C values in both leaf and xylem tissues were significantly negative in mistletoe than in its host (**Table 2**). Leaf δ^15^N was more enriched in mistletoe than in its host, while no difference in xylem δ^15^N was found between mistletoe and its host (**Table 2**).

### Macronutrient concentrations

**Figure 4:**
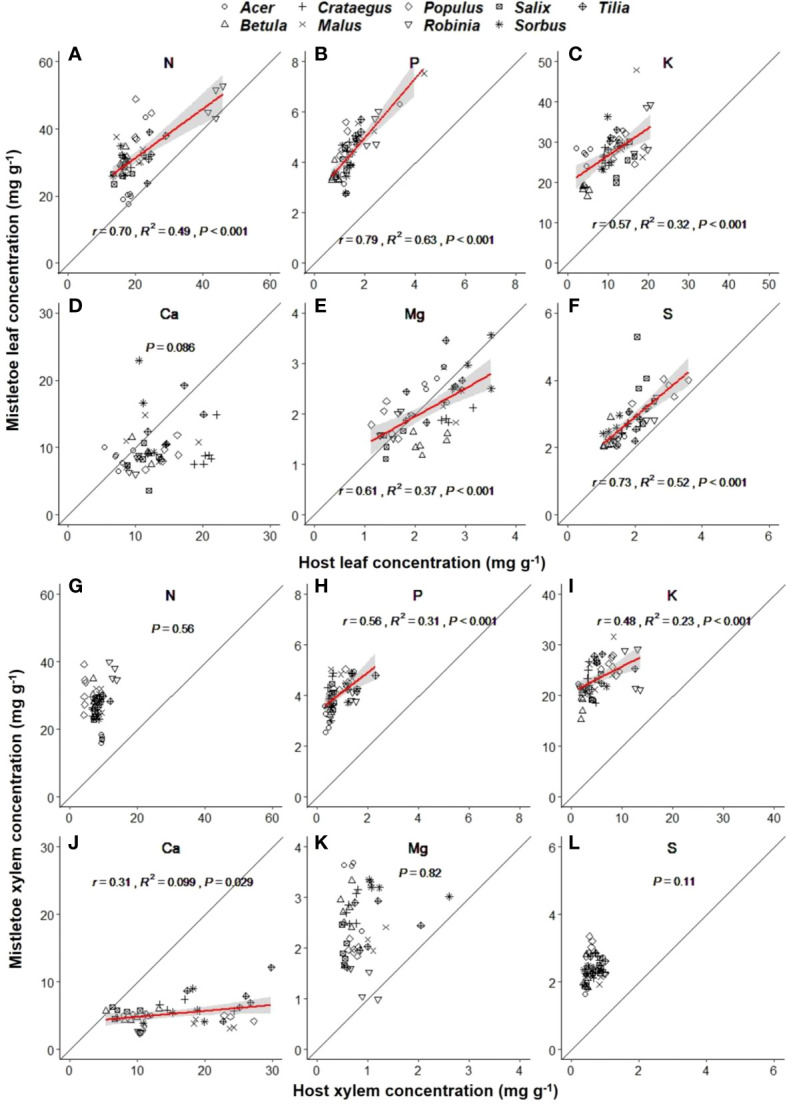
Linear relationships between mistletoe (*Viscum album* ssp. *album*) and its host for macronutrient concentrations in leaf **(A–F)** and xylem **(G–L)** tissues across nine mistletoe–host pairs in central Switzerland (indicated by different symbols). The standardized macro axis (SMA) and the 95% confidence interval are indicated with a red line and a gray band, respectively (where P<0.05). The black solid line denotes the 1:1 line. The x- and y- scale differs across the panels according to the concentrations of different elements.

**Figure 5:**
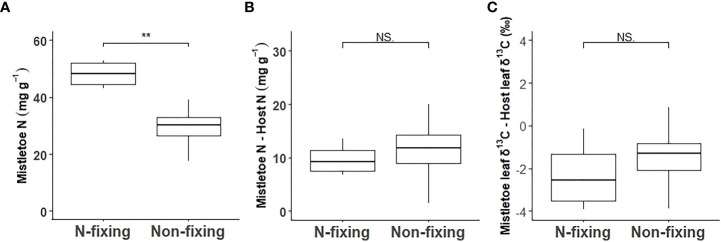
Comparison between the N-fixing host species *Robinia pseudoacacia* and the eight non-N-fixing host tree species for leaf N concentration in mistletoe (*Viscum album* ssp. *album*) **(A)** and for the difference in leaf N concentration **(B)** and in leaf carbon isotope ratio (δ^13^C) **(C)** between mistletoe and its host tree. The statistical significance of differences between N-fixing and non-N-fixing mistletoe–host pairs are given for each panel (**P<0.01; ***P<0.001, NS.: P>0.05).

Macronutrient concentrations varied significantly across the nine mistletoe–host pairs in both mistletoe and host leaf and xylem tissues, except for Ca in mistletoe leaves (**Table 1**). Strong linear relationships were found for the leaf concentrations of N (r=0.70, R^2 ^= 0.49, P<0.001), P (r=0.79, R^2 ^= 0.63, P<0.001), K (r=0.57, R^2 ^= 0.32, P<0.001), Mg (r=0.61, R^2 ^= 0.37, P<0.001) and S (R^2 ^= 0.53, P<0.001) between mistletoe and its host across of the nine mistletoe–host pairs (**Figures 4A–C, E, F**), whereas in xylem tissue positive linear correlations were only apparent for P (r=0.56, R^2 ^= 0.31, P<0.001), K (r=0.48, R^2 ^= 0.23, P<0.001), and Ca (r=0.31, R^2 ^= 0.099, P=0.029; **Figures 4H–J**). The leaf concentrations of N, P and K were significantly higher in mistletoe than in its host, and a similar tendency was found for N, P, K, Mg and S concentrations in xylem among the nine mistletoe–host pairs (**Table 2**). Further, the leaf N concentration of mistletoe grown on the N-fixing host species (*R. pseudoacacia*) was significantly higher than that for mistletoe on the non-N-fixing host species (**Figure 5A**). However, the difference in leaf N concentrations and leaf δ^13^C between mistletoe and its host did not differ between mistletoe–N-fixing host pairs and pairs with non-N-fixing hosts (**Figures 5B, C**).

### NSC–N–P stoichiometry

**Figure 6:**
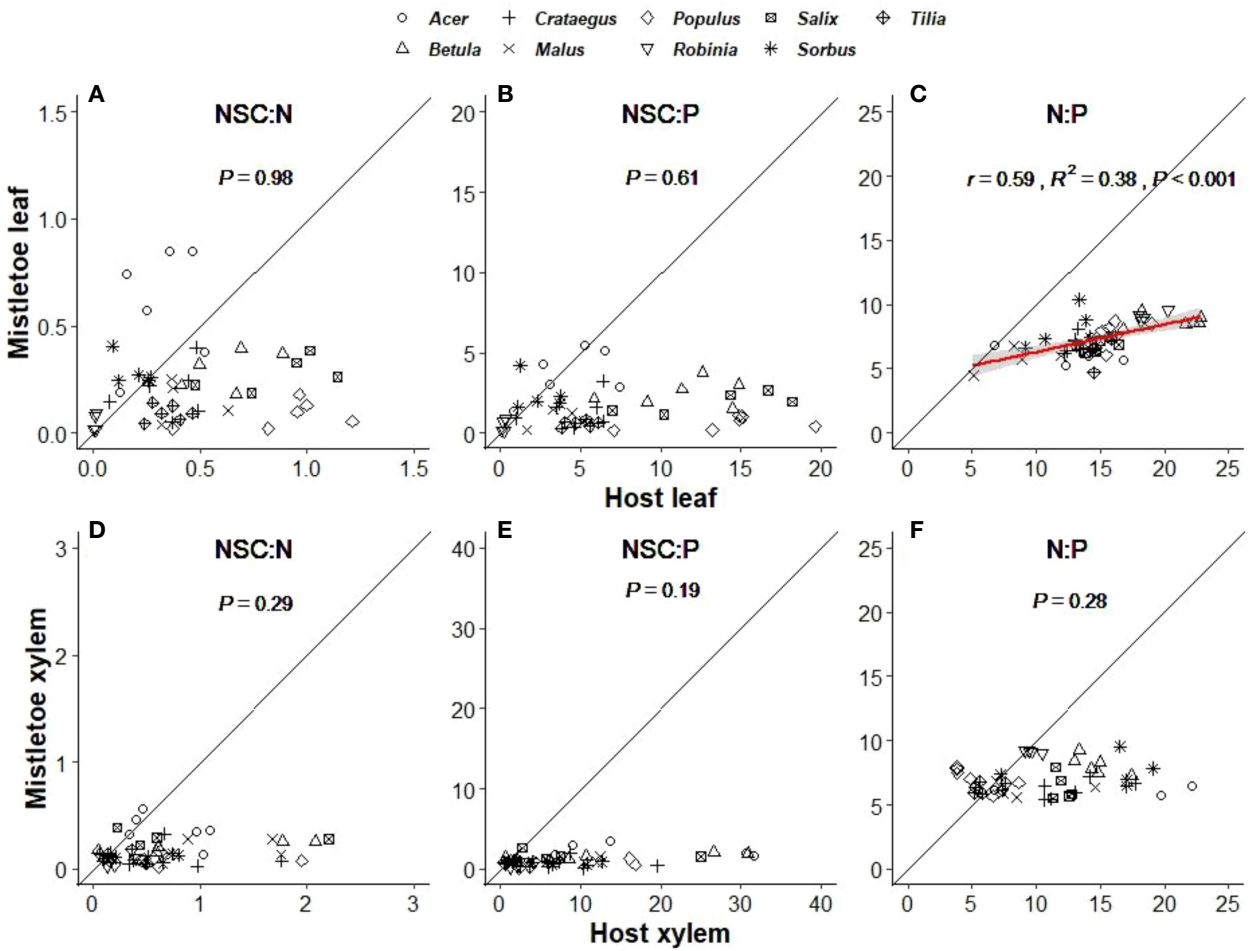
Linear relationships between mistletoe (*Viscum album* ssp. *album*) and its host for stoichiometric ratios related to concentrations of total non-structural carbohydrates (NSC), nitrogen (N) and phosphorous (P) in leaf **(A–C)** and xylem **(D–F)** tissues across nine mistletoe–host pairs in central Switzerland (indicated by different symbols). The standardized major axis (SMA) and the 95% confidence interval are indicated with a red line and a gray band, respectively (where P<0.05). The black solid line denotes the 1:1 line. The x- and y- scale differs across the panels according to the values of each variable.

Stoichiometry related to NSCs, N and P varied significantly across the nine mistletoe–host pairs in both host and mistletoe leaf and xylem tissues, except for NSC:N and NSC:P in host xylem (**Table 1**). However, a linear correlation between the values mistletoe and its host was only found for leaf N:P (r=0.59, R2 = 0.38, P<0.001; **Figure 6C**), not for other stoichiometry ratios between mistletoe and its host (**Figures 6A, B, D–F**). The stoichiometric ratios (NSC:N, NSC:P and N:P) were all significantly lower in mistletoe than in its host within each tissue type (**Table 2**).

## Discussion

### NSC concentrations in *Viscum album* ssp*. album* are not correlated with those in its hosts

No significant correlation was found for the concentration of total NSCs or individual NSC compounds between mistletoe and host leaf or xylem tissue among the nine mistletoe–host pairs (**Figure 2**). Heterotrophic carbon transfer from host to mistletoe tissues has been discussed for a long time (Schulze et al., 1984; Těšitel et al., 2010). In several previous studies stable carbon isotope ratios were used to estimate the carbon uptake of mistletoe from host tissues, and findings indicated that the heterotrophic carbon uptake varied among mistletoe species (Wang et al., 2008; Mostaghimi et al., 2021). Moreover, Giesemann and Gebauer (2022) performed a simulation study using δ^13^C, δ^18^O and δ^2^H of different mistletoe and host species to calculate the amount of heterotrophic carbon uptake. The results showed plausible differences in terms of heterotrophic carbon in C_3_-, C_4_- and CAM-hosts, as well as in different mistletoe species. Nevertheless, the current models and equations for calculating heterotrophic carbon in mistletoe are still speculative since they cannot quantify the exact carbon transfer from the host to mistletoe tissues (Bannister and Strong, 2001; Tennakoon et al., 2011). Meanwhile, even though the photosynthetic rate is found relatively lower in mistletoes compare to its host, the chlorophyll content per unit leaf area of mistletoe is higher than that of the host, suggesting that the light and potential of these plants is likely to be inhibited by some specific mechanisms (Harpe et al., 1981). Hence, the variation of NSC concentrations in mistletoe tissues among the nine mistletoe-host pairs is a compound consequence by both the heterotrophic carbon transfer and potential different photosynthetic activity rate when growing on different host trees. Moreover, among the nine mistletoe–host pairs considered in the present study, δ^13^C values in mistletoe tissues were significantly more negative (i.e. tissues were more depleted in ^13^C) than values in host tissues (i.e. leaf and xylem; **Table 2**, **Figure 3E**). This does not support the “C-parasitism hypothesis”, as carbon retrieved from the host xylem is expected to be more ^13^C-enriched compared with values in host leaves (Cernusak et al., 2004). Instead, this result may reflect the environmentally induced and species-specific differences in leaf intracellular CO_2_ concentrations (Farquhar et al., 1982; Pate, 2001). Holo-parasitic plants (fully dependent on the host for carbon) were observed to be more depleted in ^13^C (by 1.5‰), while hemiparasitic mistletoes were more enriched (by 1.2‰) compared with their hosts (Cernusak et al., 2004). These findings imply that the carbon metabolism of hemiparasitic mistletoe and its hosts may not share overlapping processes and the heterotrophic carbon provision is not the only limitation factor for the carbon status of mistletoes. In summary, our results suggest that *V. album* ssp. *album* does not rely on carbon from its broadleaf host trees and that biomass production of this species depends on both heterotrophic carbon accumulation and its own photosynthetic capacity.

### Leaf morphological consistency and adjustments in the water-relations of *Viscum album* ssp*. album* growing on different host trees

While leaf morphology differed greatly among the host tree species (**Table 1**, **Figure S1**), we did not find differences in leaf morphological traits (i.e. single leaf area, single leaf mass and LMA) of *V. album* ssp*. album* across the mistletoe–host pairs and the nine sites differing in environmental conditions (i.e. closed forest, dry slope, lakeside; **Table S1**). This is surprising since a controlled experiment conducted in a long-term irrigation forest showed that *V. album* ssp. *austriacum*, another sub-species of *V. album*, had larger leaves and a lower LMA than mistletoe growing in wetter conditions (Wang et al., 2021; Wang et al., 2022). Further, Schulze and Ehleringer (1984) found that *Phoradendron juniperinum*, another hemiparasitic mistletoe, had a 7 times higher growth rate when growing on N-fixing host trees (*Acacia greggii*) compared with values on non-N-fixing host trees (*Juniperus osteosperma*), which was mainly attributed to a 3.5 times higher N concentration in the xylem tissue of the N-fixing host. In addition to that, the morphological characteristic of host trees can also be affects by the infection severity due the increase of competitive pressure, Ozturk et al. (2022) found a significant decrease of needle dimension and stomatal size with increasing mistletoe density. It is thus likely that host species variations in nutrient availability and competitive ability can influence mistletoe morphological traits. Our mistletoe samples were all collected from the tops of the host branches or trees, and thus they had similar light conditions. Also, all the sampled trees were severely infected by the *V. album* with at least six huge clusters growing on the branches, likely leading to the observed similar morphological traits. However, all host trees in the present study were deciduous species, which may also have affected the leaf water content and morphology of mistletoe in a similar way. For example, the leaf stomata of mistletoe on deciduous hosts have been found to be 1.4-fold denser but 1.2-fold smaller in width compared with the stomata of mistletoe on evergreen hosts (Scalon et al., 2016), showing adaptation of mistletoe to the hosts.

We found stable plant water relations among the nine mistletoe–host pairs, as demonstrated by the significant correlations in δ^13^C and leaf water content between mistletoe and its hosts (**Figures 3D, E**). These results indicate that *V. album* ssp. *album* can maintain its leaf structure irrespective of changes in environmental factors, and that it regulates its physiological functions autonomously in order to survive. Leaf water content is a complex variable that is determined by the sampling time and weather conditions, as well as the water transfer between the intercellular space and the atmosphere, which might not provide straightforward evidence for evaluating the water relationship between mistletoe and its hosts. However, leaf δ^13^C values have been well studied regarding their ability to explain water use efficiency (WUE) and water transfer mechanisms between mistletoe and its hosts (Panvini and Eickmeier, 1993; Popp and Richter, 1998; Wang et al., 2021). The more negative δ^13^C values in mistletoe compared with host trees (i.e. greater depletion in ^13^C) observed here was likely caused by higher transpiration rates and lower assimilation rates, and thus a lower WUE of mistletoe tissues relative to the hosts (Wang et al., 2008; Zweifel et al., 2012; Wang et al., 2021; Griebel et al., 2022). To ensure that its own water needs are met, *V. album* has been found to maintain high stomatal conductance under drought conditions where host trees exhibit stomatal closure to reduce water loss (Zweifel et al., 2012), demonstrating how this hemiparasite is able to prioritize its own growth over the physiological needs of the host species in stressful situations (Schulze and Ehleringer, 1984). Furthermore, Scalon et al. (2016) investigated the mistletoe *Passovia ovata* growing on both evergreen and deciduous hosts and reported that mistletoe on deciduous hosts had a significantly higher WUE in summer than during the dormant season, but that WUE did not change with season when the mistletoe parasitized the evergreen hosts. These results of deciduous mistletoe-host pairs in growing season are consistent with our findings.

### Macronutrient concentrations in mistletoe are determined by the corresponding concentrations in its host

The higher transpiration rate and stomatal conductance in mistletoe than in its host have been regarded as a mistletoe strategy to easily absorb water and nutrients from the host xylem to maintain higher macronutrient concentrations than in host tissues (Ture et al., 2010; Chen et al., 2013; Scalon et al., 2013). This was confirmed by our observations (**Table 2**, **Figure 4**). Further, several studies have indicated that the host cannot quickly compensate for the macronutrients (i.e. N, P, K) consumed by the mistletoe through greater uptake from the soil, resulting in higher concentrations for these elements in mistletoe tissues than in the host (Bowie and Ward, 2004; Lamien et al., 2006; Hosseini et al., 2007). Likewise, for K the ionic form K^+^ plays an important role in regulating stomatal conductance and osmosis-related processes, making it possible for mistletoe to maintain a lower water potential (Lo Gullo et al., 2012; Le et al., 2016a) and to keep high stomatal conductance in drought conditions (Zweifel et al., 2012). We found that the difference in element concentrations was larger between mistletoe xylem and host xylem than between mistletoe leaves and host leaves (**Table 2**). We expect that this is mainly due to the absence of a phloem connection between mistletoe and its hosts, as the lack of a translocation system through phloem sap ultimately leads to a greater accumulation of macronutrients in the xylem tissue of mistletoe. Additionally, we found that most macronutrient concentrations showed positive correlations between mistletoe and host leaves, except for Ca, suggesting that the nutrient status of mistletoe leaves is determined by the concentrations in host leaves (Schulze et al., 1984; Okubamichael et al., 2011).

We found a significantly higher N concentration in mistletoe on N-fixing hosts than on non-N-fixing hosts (**Figure 5A**), and a highly significant correlation of leaf δ^15^N between mistletoe and host (R^2 ^= 0.94, P<0.001) across the nine mistletoe–host pairs (both N-fixing and non-N-fixing hosts, **Figure 3F**), indicating a high N dependency of mistletoe on its hosts. However, the difference in leaf N concentrations and δ^13^C values between mistletoe and host leaves (i.e. the value in mistletoe minus the value in the corresponding host) did not differ significantly between N-fixing and non-N-fixing hosts (**Figures 5B, C**). Scalon and Wright (2015) investigated the nitrogen relationship between mistletoe and its hosts, covering 168 mistletoe–host pairs on a global scale, and did not find any evidence for the “N-parasitism” hypothesis, except in some mimic mistletoe species occurring in the tropics, which adjust their N-absorption mechanisms when growing on N-fixing hosts by imitating the morphological traits of their host trees. Similarly, our study does not support the “N-parasitism” hypothesis for *V. album* ssp. *album*. More N was available in in N-fixing mistletoe–host pairs than in non-N-fixing pairs (**Figure 5A**), but there was not a larger difference in leaf δ^13^C or leaf N concentration between mistletoe and its host (**Figures 5B, C**) across the nine mistletoe–host pairs, suggesting that this mistletoe species does not change its N-absorption strategy depending on host N availability. We therefore speculate that N is not a factor limiting the growth and survival of *V. album* ssp. *album*, as the N concentration in mistletoe is apparently directly determined by the corresponding level in its host.

### C-N-P stoichiometry in mistletoe-pairs

Our results indicated that the ratio of NSC:N and NSC:P in host xylem tissue did not vary with host species identity or site (**Tables 1**, **S2**). However, plant stoichiometry has been reported to be affected by soil nutrient availability (Li et al., 2014; Chen and Chen, 2021). Hence, the relatively constant stoichiometric ratios of host xylem tissue may imply that, although the site characteristics for the sampling sites were quite different (i.e. closed forest, dry slope and lakeside), the soil nutrient conditions may be similar. NSC:N and NSC:P were significantly higher in host tissues than in mistletoe tissues, which was mainly caused by the accumulation of N and P in the mistletoe tissues (**Figures 4A, B**). Moreover, we found a significant linear relationship for leaf N:P between mistletoe and its host across the nine mistletoe–host pairs (**Figure 6C**). Normally, plant N:P is determined by soil nutrient conditions, transpiration rate and growth demand (Elser et al., 2003; Tessier and Raynal, 2003; Reich and Oleksyn, 2004; Hogan et al., 2021). However, *V. album* ssp. *album* does not connect with the soil, and it also lacks a connection with the phloem-sap translocation channels of its hosts (Lamont, 1983; Smith and Gledhill, 1983). This special structure resulted in N and P accumulations in mistletoe xylem in our study (**Table 2**, **Figures 4**, **5**). We found a positive correlation for N:P between mistletoe leaves and host leaves but not between mistletoe xylem and host xylem across the nine mistletoe–host pairs (**Figures 6C, F**), which can be interpreted as a self-regulation mechanism of *V. album* ssp. *album* associated with photosynthesis, respiration and transpiration processes that occur in leaves, leading to a stable stoichiometry with nutrient re-allocations (Tang et al., 2019). N and P are the most important elements involved in plant physiological processes such as photosynthesis (Reich et al., 2009; Domingues et al., 2010). As the hemiparasite mistletoe *V. album* ssp. *album* photosynthesizes autonomously, its photosynthetic process may involve a mechanism to balance the N:P ratio in leaves in relation to the ratio in hosts (**Figure 5C**), while other elements may be solely affected by the leaf transpiration process from host to mistletoe (Lo Gullo et al., 2012; Urban et al., 2012).

## Conclusion

This assessment of nine mistletoe–host pairs revealed a general pattern of mistletoe–host relationships in terms of carbon, water and nutrients. In line with our first hypothesis, the carbon status of *V. album* ssp. *album* was not determined by the corresponding status of its host, which suggests a combined effect of both heterotrophic carbon transfer and self-photosynthetic capacity. Similarly, the mistletoe leaf morphology did not change with host species identity across the nine mistletoe–host pairs. Consistent with our second hypothesis, we found a strong correlation for leaf δ^13^C and leaf water content between mistletoe and host under different growth conditions. This provides evidence of sensitive uptake adjustments of *V. album* ssp. *album* in response to different nutrient, light and soil-water availabilities. In terms of nutrient relationships, macronutrient concentrations showed significant positive linear relationships between mistletoe and its host, which supports our third hypothesis that the nutrient concentrations in mistletoe are dependent on the corresponding concentrations in its host. The macronutrient concentrations were higher in mistletoe than in its host, which is a result of nutrient accumulation in mistletoe due to continuous uptake from the host and the absence of translocation systems through phloem sap. Mistletoe leaves showed greater N accumulations in N-fixing mistletoe–host pairs than in the non-N-fixing pairs, which supports our fourth hypothesis. Meanwhile, the observed positive correlation for leaf N:P between mistletoe and its host illustrates that the photosynthetic process of *V. album* ssp. *album* may balance its N:P ratio to maintain optimal photosynthetic activity.

## Data availability statement

The raw data supporting the conclusions of this article will be made available by the authors, without undue reservation.

## Author contributions

ML, AR and AW planned the sampling assessment. AW and ML conducted the field sampling work. AW conducted experiment work and analyzed the data. AW and ML wrote the manuscript. MLe, AG, AR, AB, LY and MLi revised the manuscript. All authors contributed to the article and approved the submitted version.
