# Supplementary material for: Water status and macronutrient concentrations, but not carbon status, of Viscum album ssp. album are determined by its hosts: a study across nine mistletoe–host pairs in central Switzerland
Source: Front Plant Sci. 2023 May 8;14:1142760. doi: 10.3389/fpls.2023.1142760 (PMC10200922; doi:10.3389/fpls.2023.1142760)
Supplement: Supplementary file 1 [file DataSheet_1.docx]

**Appendix: Supporting figures and tables**


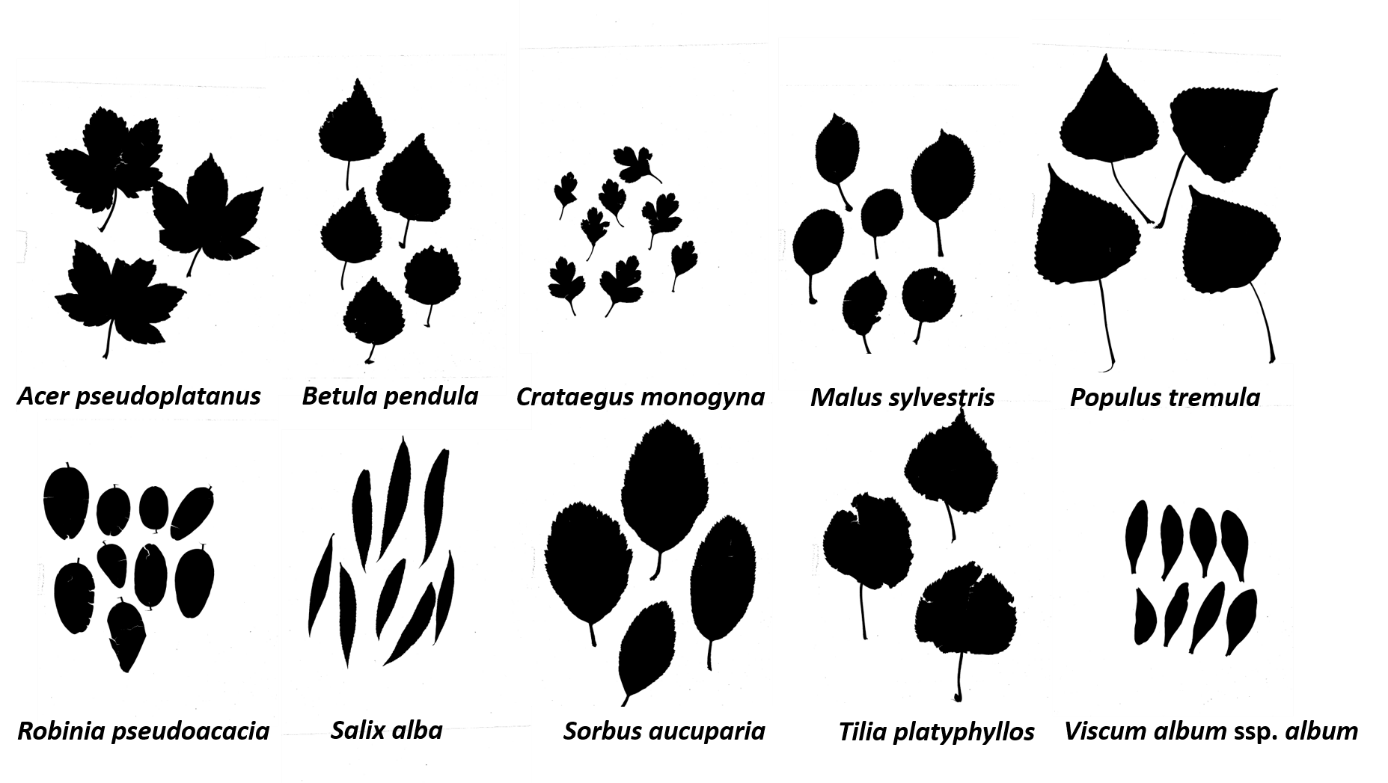


**Figure S1:** Images of the leaves of the nine broadleaf deciduous hosts and *Viscum album* ssp. *album*. The leaves shown here were randomly selected from the samples and scanned in the lab.

**Table S1:** Site information and growth indicators for the nine studied host species. *Viscum album* ssp*. album* was the mistletoe species in all pairs.

| Host  species | Height  (m) | DBH  (cm) | Location | Latitude  & longitude | Site conditions | Elevation  (m a.s.l.) | Precipitation  (mm) |
| --- | --- | --- | --- | --- | --- | --- | --- |
| *Acer*  *pseudoplatanus* | 5.8±0.1 | 10.2±0.6 | Innertkirchen | 46°42'32" N,  8°14'29" E | Closed forest | 586.4 | 610.3 |
| *Tilia*  *platyphyllos* | 6.4±0.3 | 9.9±0.7 | Brienzwiler | 46°45'4" N,  8°5'55" E | Closed forest | 663.4 | 630.4 |
| *Crataegus*  *monogyna* | 4.9±0.3 | 9.8±0.4 | Brienzwiler | 46°45'4" N,  8°5'55" E | Closed forest | 663.4 | 630.4 |
| *Robinia*  *pseudoacacia* | 7.1±0.2 | 10.4±0.5 | Brienzwiler | 46°45'4" N,  8°5'55" E | Closed forest | 663.4 | 630.4 |
| *Sorbus*  *aucuparia* | 5.9±0.3 | 11.2±0.7 | Brienzwiler | 46°45'4" N,  8°5'55" E | Closed forest | 663.4 | 630.4 |
| *Salix*  *alba* | 5.6±0.3 | 10.6±0.4 | Brienz | 46°45'20" N,  8°0'28" E | Lakeside | 675.1 | 720.8 |
| *Malus*  *sylvestris* | 6.6±0.8 | 9.7±0.3 | Ebligen | 46°45'13" N,  7°59'38" E | Dry slope | 578.2 | 580.6 |
| *Populus*  *tremula* | 6.9±0.4 | 9.6±0.5 | Aaregg Brienz | 46°44'32" N,  8°2'56" E | Lakeside | 445.2 | 710.6 |
| *Betula*  *pendula* | 7.3±0.5 | 10.7±0.2 | Auslikon | 47°20'37" N,  8°47'34" E | Lakeside | 512.6 | 732.9 |

**Table S2:** One-way ANOVA results for the effects of sampling site (*n*=6) on different variables: total non-structural carbohydrate (NSC), sugar and starch concentrations, single leaf area and mass, leaf dry mass per unit leaf area (LMA), leaf water content, nutrient concentrations (nitrogen [N], phosphorus [P], potassium [K], calcium [Ca], magnesium [Mg] and sulfur [S]), and element stoichiometry in leaves and xylem tissues of mistletoe and host species. The number of degrees of freedom (DF) and F-values are given.

|  | Variable | DF | Host Leaf | Mistletoe leaf | Host xylem | Mistletoe xylem |
| --- | --- | --- | --- | --- | --- | --- |
| Available carbon  (% dry matter) | **NSC** | 5 | 2.6 | 10.2*** | 1.8 | 10.2*** |
|  | **Sugars** | 5 | 2.5 | 5.3*** | 2.4 | 3.1 |
|  | **Starch** | 5 | 3.9** | 4.6** | 1.6 | 9.3*** |
| Isotope ratio (‰) | **δ^13^C** | 5 | 3.8** | 2.5* | 4.2*** | 3.01* |
|  | **δ^15^N** | 5 | 14.9*** | 11.5*** | 16.7*** | 10.5*** |
| Leaf water content (%) | | 5 | 8.1*** | 2.6 | NA | NA |
| Single leaf area (cm^2^) | | 5 | 14.5*** | 1.9 | NA | NA |
| Single leaf mass (g) | | 5 | 3.1* | 2.1 | NA | NA |
| LMA (cm^2^ g^-1^) | | 5 | 16.3** | 1.6 | NA | NA |
| Nutrient  concentrations  (mg g^-1^) | **N** | 5 | 4.6** | 9.4*** | 3.9** | 11.5*** |
|  | **P** | 5 | 5.7*** | 6.9*** | 2.7* | 5.5*** |
|  | **K** | 5 | 24.5*** | 7.5*** | 10.7*** | 5.4*** |
|  | **Ca** | 5 | 6.8*** | 2.1 | 6.9*** | 4.2** |
|  | **Mg** | 5 | 19.2*** | 12.0*** | 2.8* | 16.9*** |
|  | **S** | 5 | 24.0*** | 17.0*** | 4.3* | 5.9*** |
| Stoichiometry | **NSC:N** | 5 | 3.9** | 14.2*** | 0.8 | 14.1*** |
|  | **NSC:P** | 5 | 6.1*** | 9.7*** | 2.4 | 10.7*** |
|  | **N:P** | 8 | 22.0*** | 8.3*** | 9.6*** | 6.5*** |

**Note *P<0.05, **P<0.01, ***P<0.001**

**Table S3:** Results of the two model approaches (fixed and fixed & random) used to test the effects of each variable related to carbon, water and nutrients on the leaves of each mistletoe–host pair from six different sampling sites. Estimate±SE, P-value and R^2^ for both models are given to indicate differences.

| Variables | Estimate±SE | P-value | *R*^2^ (fixed) | *R*^2^ (fixed & random) |
| --- | --- | --- | --- | --- |
| NSC | 0.56±0.27 | 0.06 | 0.08 | 0.08 |
| Sugars | 0.59±0.97 | 0.55 | 0.007 | 0.12 |
| Starch | 0.18±0.15 | 0.24 | 0.03 | 0.04 |
| δ^13^C | 0.58±0.11 | <0.001 | 0.28 | 0.54 |
| δ^15^N | 0.52±0.10 | <0.001 | 0.31 | 0.61 |
| N | 0.04±0.06 | 0.52 | 0.01 | 0.02 |
| P | 0.28±0.09 | 0.09 | 0.15 | 0.53 |
| K | 0.36±0.12 | <0.001 | 0.15 | 0.29 |
| Ca | 1.40±0.69 | 0.19 | 0.06 | 0.19 |
| Mg | -0.02±0.09 | 0.78 | 0.01 | 0.44 |
| S | 0.02±0.08 | 0.76 | 0.02 | 0.21 |
| Fe | -0.01±0.02 | 0.56 | 0.006 | 0.23 |
| Mn | 2.58±0.44 | <0.001 | 0.47 | 0.58 |
| Al | -0.99±0.64 | 0.13 | 0.05 | 0.23 |
| Zn | 0.73±0.07 | <0.001 | 0.66 | 0.75 |
| NSC:N | 0.89±0.84 | 0.30 | 0.02 | 0.05 |
| NSC:P | 4.48±1.56 | <0.001 | 0.15 | 0.15 |
| N:P | -1.01±0.61 | 0.10 | 0.03 | 0.51 |

**Table S4:** Results of the two model approaches (fixed and fixed & random) used to test the effects of each variable related to carbon, water and nutrients on the xylem tissues of each mistletoe–host pair from six different sampling sites. Estimate±SE, P-value and R^2^ for both models are given to indicate differences.

| Variables | Estimate±SE | P-value | *R*^2^ (fixed) | *R*^2^ (fixed & random) |
| --- | --- | --- | --- | --- |
| NSC | 0.10±0.22 | 0.66 | 0.005 | 0.17 |
| Sugars | -0.64±0.25 | 0.02 | 0.16 | 0.32 |
| Starch | 0.39±0.13 | 0.004 | 0.16 | 0.22 |
| δ^13^C | 0.63±0.11 | <0.001 | 0.40 | 0.50 |
| δ^15^N | 0.93±0.04 | <0.001 | 0.94 | 0.95 |
| Leaf water content | 1.14±0.32 | <0.001 | 0.13 | 0.61 |
| Leaf area | -3.33±1.22 | 0.009 | 0.01 | 0.56 |
| LMA | -0.10±1.15 | 0.93 | <0.001 | 0.16 |
| N | 0.67±0.10 | <0.001 | 0.49 | 0.49 |
| P | 0.54±0.06 | <0.001 | 0.63 | 0.68 |
| K | 0.35±0.07 | <0.001 | 0.22 | 0.64 |
| Ca | 0.13±0.10 | 0.19 | 0.02 | 0.61 |
| Mg | 0.39±0.11 | 0.0012 | 0.15 | 0.58 |
| S | 0.35±0.10 | 0.0014 | 0.22 | 0.55 |
| Fe | -0.12±0.13 | 0.37 | 0.02 | 0.28 |
| Mn | 0.75±0.13 | <0.001 | 0.46 | 0.63 |
| Al | -0.40±0.53 | 0.46 | 0.01 | 0.21 |
| Zn | -0.06±0.27 | 0.82 | 0.01 | 0.13 |
| NSC:N | 0.21±0.26 | 0.41 | 0.02 | 0.17 |
| NSC:P | 0.78±0.65 | 0.24 | 0.03 | 0.18 |
| N:P | 1.84±0.29 | <0.001 | 0.35 | 0.68 |
